# Small bowel GIST with hemorrhagic shock diagnosed by capsule endoscopy and double‐balloon endoscopy, angiography‐guided hemostasis, and laparoscopic‐assisted resection

**DOI:** 10.1002/ccr3.4240

**Published:** 2021-05-15

**Authors:** Hideki Kogo, Kimiyoshi Shimanuki, Toshiyasu Iwao, Hiroshi Yoshida

**Affiliations:** ^1^ Department of Surgery Nippon Medical School Tama‐Nagayama Hospital Tokyo Japan; ^2^ Department of Surgery Aizu Central Hospital Fukushima Japan; ^3^ Department of Gastroenterology Aizu Central Hospital Fukushima Japan; ^4^ Department of Gastrointestinal and Hepato‐Biliary‐Pancreatic Surgery Nippon Medical School Tokyo Japan

**Keywords:** angiography, capsule endoscopy, hemorrhagic small bowel gastrointestinal stromal tumor

## Abstract

Small bowel tumors presenting with hemorrhagic shock require urgent treatment with angiographic embolization to achieve hemostasis. Capsule endoscopy and double‐balloon endoscopy are useful for localizing the tumor, diagnosis, and guiding surgery.

## Graphical abstract

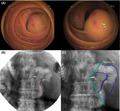

## 1 INTRODUCTION

This is a case of hemorrhagic small bowel GIST diagnosed by capsule endoscopy and double‐balloon endoscopy. Endovascular treatment was possible after abdominal angiography to visualize the specific bleeding site clearly, with coil embolization to achieve hemostasis. Further treatment by laparoscopic‐assisted partial resection of small bowel was successful.

Small intestinal bleeding is difficult to diagnose. In many cases, hemostasis cannot be achieved easily. The etiology of small bowel bleeding in patients age >40 years varies widely. Common causes include vascular lesions, such as angioectasia, tumors, ulceration from nonsteroidal anti‐inflammatory drugs, and Dieulafoy lesions.^1^, ^2^ The treatment plan depends on the etiology. Recent advances in capsule endoscopy and double‐balloon endoscopy are recommended for diagnosis and treatment.^3^ In some cases, endovascular treatment or surgical resection may be necessary.^4^, ^5^ In this case study, capsule endoscopy, double‐balloon endoscopy, angiography, and laparoscopic‐assisted surgery were used to treat small intestinal bleeding.

## 2 CASE PRESENTATION

A 66‐year‐old Japanese woman presented to the emergency room with melena for 3 days. She had a history of diabetes, hypertension, cerebral infarction, and angina. Gastrointestinal bleeding is suspected, and she was admitted to the hospital for further investigation and treatment.

On admission, vital signs were as follows: temperature, 36.2°C; blood pressure, 118/70 mm Hg; pulse, 84 beats per min; respiratory rate, 19 breaths per min; and oxygen saturation, 97%; she was breathing ambient air. Consciousness was clear, and physical examination showed pale palpebral conjunctiva; the abdomen was flat and soft without tenderness, and no mass was palpable. Laboratory evaluation was significant for hemoglobin at 4.3 g/dL and severe anemia with blood urea nitrogen at 33.6 mg/dL.

**FIGURE 1:**
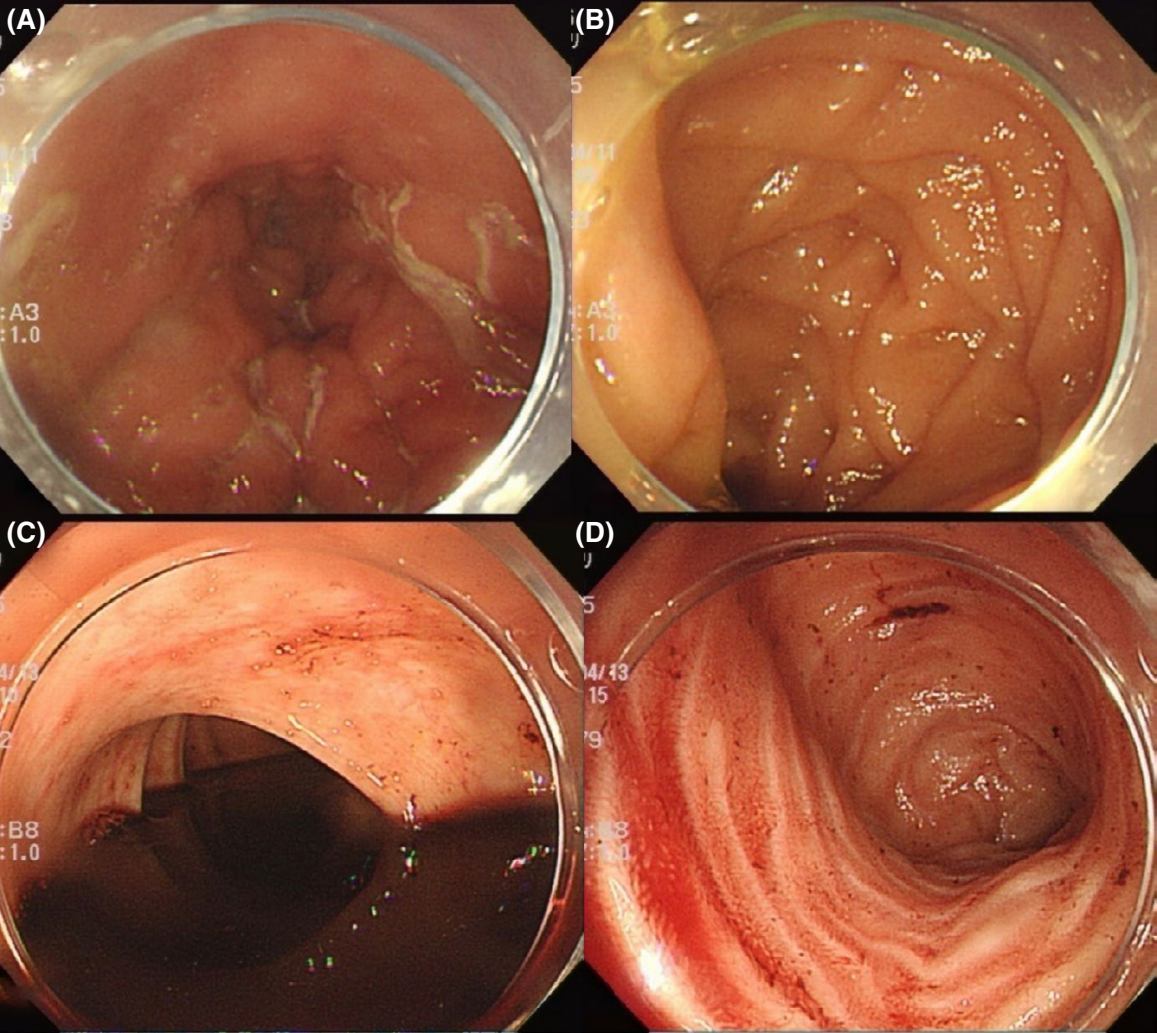
Emergency endoscopy. An upper GI endoscopy showing no bleeding lesions (A, B). A lower GI endoscopy showing no lesions (C). There was a small amount of coagula in the terminal ileum (D)

An emergent upper endoscopy revealed no vascular abnormalities, mass lesions, or ulcerative lesions as the potential source of bleeding (Figure 1A, B). A lower GI endoscopy revealed no lesions. However, altered blood was seen in the colon (Figure 1C) and terminal ileum (Figure 1D), suggesting bleeding from the small intestine.

**FIGURE 2:**
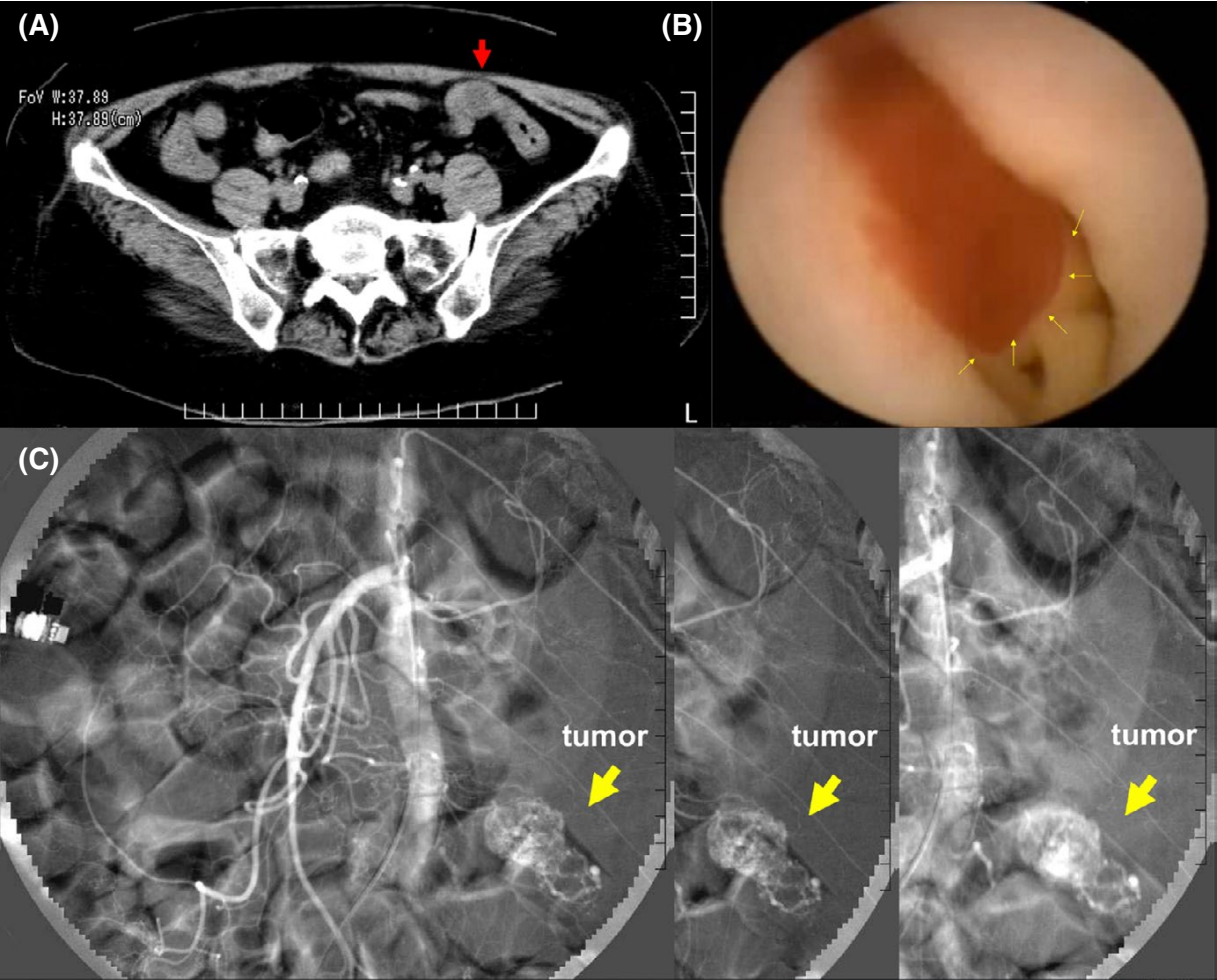
A, Abdominal computed tomography scan. CT scan of the abdomen showing a mass lesion. B, Capsule endoscopy. Capsule endoscopy showing active bleeding from the tumor in the upper small bowel. C, Abdominal angiography. A contrast agent from the superior mesenteric artery indicating a vascular tumor in the jejunal artery branch. Embolization with coils was performed in the imaged area

After admission, there was no hemorrhage, and the anemia improved with blood transfusion. On hospital day 5, however, a large amount of melena was observed and her blood pressure was 68/40 mm Hg. Computed tomography of the abdomen showed a mass lesion as (Figure 2A), but the exact location and qualitative diagnosis were difficult to determine. The differential diagnoses considered were a small intestinal tumor, gastrointestinal stromal tumor (GIST), inflammatory disease, and vascular abnormalities. The initial treatment considered was hemostasis by double‐balloon small bowel endoscopy.

A capsule endoscopy was performed on hospital day 7 to determine whether the oral or transanal approach was optimal until the patient recovered to a condition that would allow her to tolerate a small bowel endoscopy. The results of the capsule endoscopy were revealed on hospital day 9. At 1 hour and 4 minutes into the capsule endoscopy examination, active bleeding was observed, which seemed to be in the upper small bowel (Figure 2B). Thus, an emergency angiogram of the abdomen was performed on hospital day 9 for hemostasis.

A contrast agent from the superior mesenteric artery (SMA) indicated a vascular tumor in the jejunal artery branch. There were no other abnormal vessels in other parts of the body that appeared to be the source of bleeding. The patient underwent embolization with coils in the imaged area (Figure 2C), and hemostasis was successful. There was no postoperative melena, and the anemia was improved.

After the patient's general condition improved, she underwent an oral double‐balloon small intestinal endoscopy on day 13. A 40‐mm submucosal tumor (SMT) was detected 80 cm from the dental line. A GIST with erythematous dilated vessels and erosions at the apex was suspected, and surgical resection was scheduled.

**FIGURE 3:**
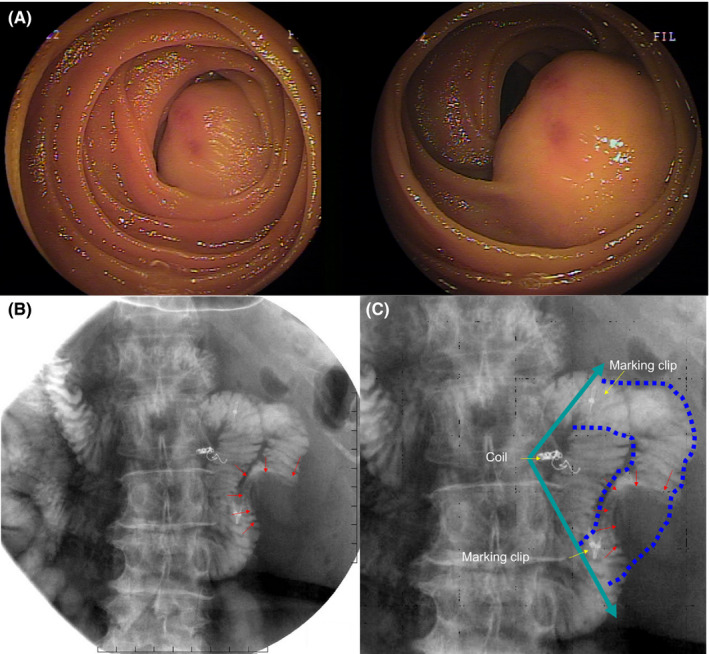
A, Double‐balloon endoscopy. Oral double‐balloon small intestinal endoscopy was performed on day 13. SMT was detected 80 cm from the dental line. Preoperatively marking clips were inserted on the oral and anal sides. B, Small bowel imaging. Small bowel imaging showing the tumor, both the marking clips, and the embolic coils. C, Expected resection area. The planned surgical resection line was fan‐shaped as shown in the figure

Preoperatively marking clips were inserted on the oral and anal sides (Figure 3A). This perspective was observed on the small bowel imaging study that showed both the marking clips and the embolic coils (Figure 3B). The resection area was planned based on this illustration. The surgical plan was to use intraoperative imaging to mark the coils and clips to resect the intestinal tract, resembling a fan‐shaped excision line (Figure 3C).

**FIGURE 4:**
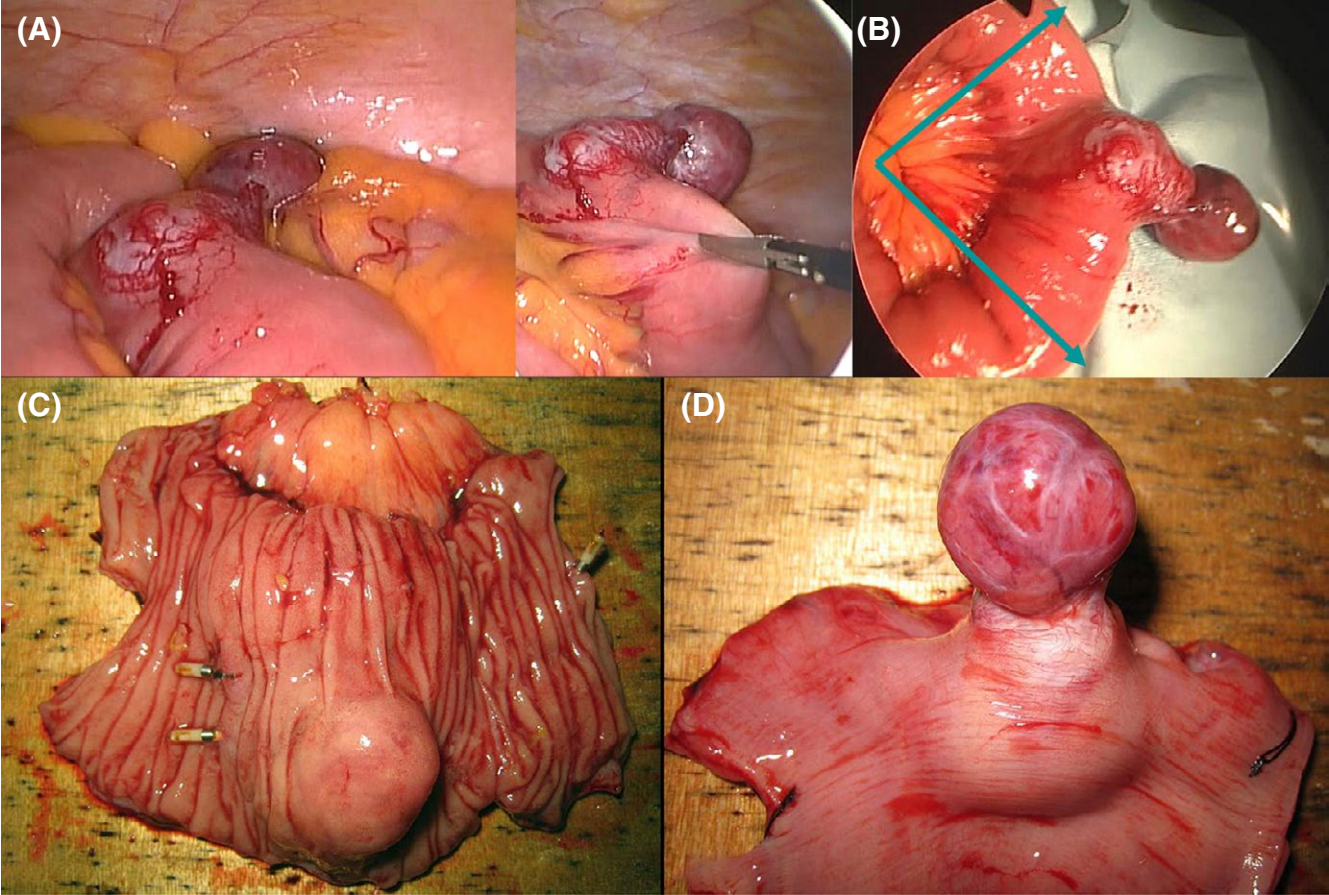
A, Laparoscopic view. Laparoscopic partial resection of the small intestine was performed. The tumor was protruding outside the intestinal wall. B, Surgical portrait. We resected the tumor using clips and coils. Resected specimen C, lumen, D, serosal site. The tumor was 45‐mm long whitish solid mass with shallow erosions protruding outside the intestinal wall

On day 32, the patient underwent a laparoscopic partial resection of the small intestine. The tumor was protruding outside the intestinal wall. Therefore, the planned intraoperative imaging was not needed (Figure 4A). The port in the wound was extended to enable lifting the small intestinal tumor out of the body. With guidance from the clips and coils, partial resection of the small intestine was performed (Figure 4B), and then, the small intestine was anastomosed. The tumor was a 45‐mm long, whitish solid mass with shallow erosions protruding outside the intestinal wall (Figure 4C, D). No bleeding or necrosis was evident. The remaining surgery was uneventful, and the patient's postoperative recovery was normal.

**FIGURE 5:**
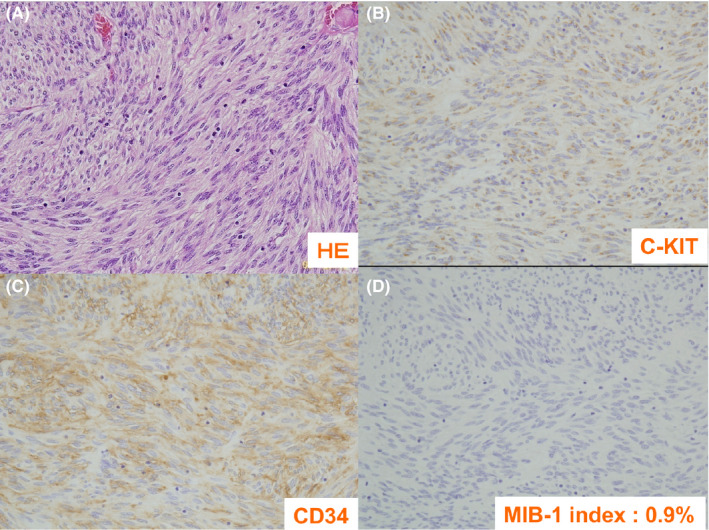
Pathological findings. HE staining showing spindle‐shaped, irregularly enlarged cells in bundles (A), and immunostaining was positive for c‐Kit (B). The rate of CD34 was positive (C), and MIB‐1‐positive findings were 0.9% (D).cell pattern with myxoid stroma (H&E). C, Tumor cells weakly immunoreactive for c‐kit (CD117) D

On pathologic examination, hematoxylin and eosin staining showed spindle‐shaped, irregularly enlarged cells in bundles, and immunostaining was positive for c‐Kit. The rate of CD34 was positive, S‐100 was negative, and MIB‐1‐positive findings were 0.9%; therefore, the tumor was classified in the low‐risk group (Figure 5A‐D).

## 3 DISCUSSION

The epidemiology of GIST is described as follows: mean age at diagnosis, 63 years; male‐to‐female ratio, 54:46; organs in which this tumor occurs are the stomach (51%), small intestine (36%), large intestine (7%), rectum (5%), and esophagus (1%). The 5‐year survival rate is 45%.^6^ Treatment is based on resection.

The distribution of clinical presentation is as follows: gastrointestinal bleeding, abdominal distention, and abdominal discomfort. Further, 15%‐30% cases have been reported to be asymptomatic.^7^, ^8^

An algorithm for the treatment of small intestinal bleeding is set out in the guidelines. A distinction is made between cases with and without shock. In cases of small bowel bleeding with shock, early angiography is recommended.^1^ In this case, there was a period of time when the tumor bleeding had stopped and the patient was out of shock. Therefore, during that stable period, capsule endoscopy was first performed to diagnose the disease and its location.^9^ The capsule endoscope revealed a GIST in a bleeding state. In this case, surgery was possible to resect a localized small bowel GIST. However, because the patient had a small intestinal GIST presenting with hemorrhagic shock, the diagnosis and treatment took time, and several diagnostic and treatment modalities were required. Ultimately, GIST of the small intestine was diagnosed by capsule endoscopy and double‐balloon endoscopy, with melena as a presenting symptom and treatment by laparoscopic surgery to resect the tumor. A minimally invasive capsule endoscope was useful in estimating the site of bleeding and determining the approach route for a double‐balloon endoscope.^10^ Double‐balloon endoscopy is challenging to perform in patients with unstable vital signs, such as in this case. Therefore, hemostasis achieved with angiography was more useful.^11^, ^12^, ^13^

Intraoperative imaging showed that the surgical resection site could be planned using coils and marking clips as indicators, which led to a laparoscopic‐assisted partial resection of the small bowel. A few reports have documented emergency surgery for hemorrhagic small bowel GIST; in this case, however, it was worthwhile to stabilize the patient first and then perform a curative resection of the tumor in minimally invasive surgery.

## 4 CONCLUSION

Diagnosis of small bowel GIST is possible using a combination of a capsule and double‐balloon endoscopy, depending on the patient's condition. Furthermore, with angiographic examination and treatment, hemostasis of the small bowel GIST was successful, subsequently allowing a laparoscopic‐assisted resection of the tumor to be performed.

## CONFLICT OF INTEREST

The authors declare that they have no competing interests.

## AUTHOR CONTRIBUTIONS

HK: drafted the manuscript. HT and KS: performed the operation. TI: performed the endoscopy. HT, KS, TI, and HY: revised the manuscript. HK, KS, TI, and HY: read and approved the final manuscript.

## CONSENT

Informed consent was obtained from the patient for the publication.

## ETHICS APPROVAL AND CONSENT TO PARTICIPATE

No ethical issue in reporting of this case.

## Data Availability

Data sharing is not applicable to this case report type article as no new data were created or analyzed in this study.
